# Effective dissecting range and prognostic significance of lateral pelvic lymph node dissection for middle-low rectal cancer patients with lateral pelvic lymph node metastasis: Results of a large multicenter lateral node collaborative group in China

**DOI:** 10.3389/fonc.2022.916285

**Published:** 2022-08-12

**Authors:** Sicheng Zhou, Jianqiang Tang, Jianwei Liang, Zheng Lou, Wei Fu, Bo Feng, Yingchi Yang, Yi Xiao, Qian Liu

**Affiliations:** ^1^ Department of Colorectal Surgery, National Cancer Center/National Clinical Research Center for Cancer/Cancer Hospital, Chinese Academy of Medical Sciences and Peking Union Medical College, Beijing, China; ^2^ Department of Colorectal Surgery, The first affiliated hospital, Navy Medical University, Shanghai, China; ^3^ Department of General Surgery, The Affiliated Hospital of Xuzhou Medical University, Xuzhou, China; ^4^ Department of General Surgery, Ruijin Hospital, Shanghai Jiao Tong University School of Medicine, Shanghai, China; ^5^ Department of General Surgery, Beijing Friendship Hospital, Capital Medical University, Beijing Key Laboratory of Cancer Invasion and Metastasis Research and National Clinical Research Center of Digestive Diseases, Beijing, China; ^6^ Department of General Surgery, Peking Union Medical College Hospital, Peking Union Medical College, Chinese Academy of Medical Sciences, Beijing, China

**Keywords:** lateral pelvic lymph node, lateral pelvic lymph node metastasis, rectal cancer, prognosis, surgical dissection

## Abstract

**Background:**

Lateral pelvic lymph node (LPN) metastasis causes increased lateral local recurrence and poor prognosis. We aimed to investigate the prognostic significance and effective range of dissection for the LPN dissection (LPND) in rectal cancer patients with LPN metastasis.

**Materials and methods:**

Through this large, multicenter retrospective cohort study, we evaluated the therapeutic effect of LPND. From January 2012 to December 2019, 387 rectal cancer patients with clinical evidence of LPN metastasis who underwent total mesorectal excision with LPND were included in the study. According to pathological findings, they were divided into negative (n = 296) and positive (n = 91) LPN groups. Primary endpoints were 3-year overall survival (OS), recurrence-free survival (RFS), and local recurrence-free survival (LRFS).

**Results:**

The OS, RFS, and LRFS in the positive group were significantly worse than those in the negative group; However, LPN metastases were not independent prognostic risk factors for LRFS (hazard ratio [HR]: 2.42; 95% confidence interval [CI], 0.77–7.64; *P*=0.132). Among patients with pathological LPN metastases, LPN metastases to the common and external iliac arteries were independent prognostic risk factors both for OS (HR: 4.74; 95% CI, 1.74–12.90; *P*=0.002) and RFS (HR: 2.70; 95% CI, 1.16–6.29; *P*=0.021). No significant difference was observed in the 3-year OS (72.3% vs. 70.2%, *P*=0.775) and RFS rates (60.9% vs. 52.6%, *P*=0.408) between patients with metastases to the obturator or internal iliac arteries and patients at N2b stage.

**Conclusions:**

LPND may be effective in controlling local recurrence in patients with LPN metastasis but not systemic metastases. Patients with LPN metastasis limited to the internal iliac and obturator regions achieve a long-term survival benefit from LPND, and their prognoses may be comparable to those at the N2b stage. Further metastasis to the external iliac or common iliac region should be considered systemic disease, and LPND should be avoided.

**Clinical Trial Registration:**

ClinicalTrials.gov, identifier NCT04850027.

## Introduction

Following the introduction of neoadjuvant chem-oradiotherapy (nCRT) and standard surgical procedures with total mesorectal excision (TME) (1), the 5-year local recurrence (LR) rate of locally advanced rectal cancer was 5%−10% (2–4). For locally advanced rectal cancer below peritoneal reflection, the incidence of lateral lymph node (LPN) metastasis is 16%–23% (5–7), which exceeds the scope of routine TME dissection. Further, increasing evidence suggests that LPN metastasis is an important cause of LR after surgery (8). Lateral pelvic lymph node dissection (LPND), as a preventive and potential curative surgery for lateral pelvic recurrence, is still controversial worldwide (9–11). In particular, surgeons in western countries consider LPN metastasis as a systemic disease (except for internal iliac lymph nodes) and prefer to implement TME alone (10). Conversely, as per the guidelines in Japan, LPN metastasis is considered a regional metastasis that develops mainly in those with middle-low cT3-T4 rectal cancer; further, the LPND showed therapeutically beneficial effects (11). Therefore, the Japanese Society for Cancer of the Colon and Rectum (JSCCR) recommended TME plus central D3 resection and prophylactic LPND as the standard procedure for advanced low rectal cancer (12).

In the JCOG0212 large-scale clinical trial, compared with TME alone, TME with LPND could not improve the overall survival (OS), but it was particularly effective in suppressing the LR within the lateral pelvis, including the LPNs (10). Further, Kanemitus et al. reported that 1,191 patients with rectal cancer from two large-volume centers in Japan received TME with LPND; the results showed that dissection of the internal iliac and obturator lymph nodes was comparable to the treatment of superior rectal artery lymph node dissection; however, the risk of LR during the unilateral LPND was twice than that of the bilateral LPND (13). We did not specifically target patients with LPN metastases; the JCOG2012 trial excluded almost 20% of the patients with clinically suspected LPN metastasis. The therapeutic effect of LPND and its prognostic significance in patients with LPN metastasis have not yet been clarified. Therefore, we designed a large, multicenter retrospective study to evaluate the therapeutic effect of LPND on local control and survival benefit in patients with LPN metastasis; we also explored the prognostic significance and effective range of dissection for LPND.

## Materials and methods

### Patients

This was a large, multicenter retrospective cohort study. From January 2012 to December 2019, 485 rectal cancer patients with clinical evidence of LPN metastasis who underwent TME with LPND were identified in three participating institutions of the Chinese Lateral Node Collaborative Group in China (195 cases were recruited from the Cancer Hospital, Chinese Academy of Medical Sciences and Peking Union Medical College; 152 cases were recruited from the Peking University First Hospital; and 138 cases were recruited from Peking Union Medical College).

The patients were included based on the following inclusion criteria (1): clinical evidence of LPN metastasis based on magnetic resonance imaging (MRI) evaluation, (2) clinically advanced rectal cancer (cT3-T4/cN+), (3) pathologically confirmed adenocarcinoma, and (4) lower margin of the tumor located below the peritoneal reflection. The exclusion criteria were as follows: (1) presence of simultaneous distant metastases, (2) having undergone total or partial pelvic exenteration, (3) having undergone local resection or R2 resection, and (4) not having undergone standard LPND according to the JSCCR guidelines. Each of the three participating institutions received local ethical approval for the study, and the protocol was registered (NCT04850027) at ClinicalTrials.gov. Informed consent was obtained from all the patients prior to enrollment; all the study procedures were in accordance with the tenets of the Declaration of Helsinki.

### Diagnosis and treatment strategies

MRI was used to detect and evaluate the LPN metastasis; the diagnosis was based on meeting one or more of the following diagnostic criteria: (1) ≥0.5 cm in the short axis before treatment, (2) inhomogeneous or intense enhancement, and (3) irregular shape and rough edges. The details of all patients were discussed in a multidisciplinary team meeting (MDT) discussion with radiologists and medical and surgical oncologists to determine the treatment strategies for individual patients. The American Joint Committee on Cancer (AJCC) staging system (8^th^ edition) was used to perform TNM staging (14). Postoperative complications were defined as events that occurred within 30 days after the surgery and were categorized according to the Clavien–Dindo classification (15).

### LPND procedure

All chief surgeons completed at least 500 cases of laparoscopic colorectal surgery and mastered the mature LPND technique. Surgical procedures were generally similar in the three institutions, and LPND (with preservation of the autonomic nerves) was performed in accordance with the previously reported methods (16, 17). Briefly, after total mobilization of the rectum and distal rectal transection according to the TME principle, unilateral or bilateral LPND was performed appropriately according to the location of the enlarged lateral lymph nodes detected on preoperative MRI. LPNs were divided into the common iliac vessel, proximal, distal internal iliac vessel, obturator, and external iliac vessel regions (12), and the extent of LPND was defined as resection of lymphatic tissue in the entire area (described above). Unless the internal iliac artery or its branches were invaded, they were dissected by skeletonization and routinely preserved during the dissection. The autonomic and obturator nerves were carefully identified and preserved during the procedure.

### Follow-up

The indications for postoperative adjuvant chemotherapy were high-risk stages II and III, and all patients with LPN metastasis underwent adjuvant therapy. Patients were followed up through outpatient visits, with clinical examination and serum tumor markers evaluation every 3 months, along with a CT examination of the chest, abdomen, and pelvis every 6 months in the first 3 years. Three years after the surgery, the patients were followed-up every 6–12 months until November 30, 2021. During the LR events, the pelvic MRI was reviewed to determine the location of metastasis. Relapse includes LR and distant metastases; LR can be divided into central (anterior, presacral, anastomotic site, or perineal) and lateral pelvic sites. The endpoints of this study were 3-year LR-free survival (LRFS), 3-year recurrence-free survival (RFS), and 3-year OS.

### Statistical analysis

Categorical and continuous variables were compared using the chi-squared test and t-test, respectively. LRFS, RFS, and OS were assessed using the Kaplan–Meier method and compared using the log-rank test. Univariate logistic and Cox regression models were used to analyze the effects of the co-variables and to determine the risk factors. Multivariate analysis was performed using co-variables with a significant effect (*P* < 0.05) in the univariate analysis, and the effect of each variable was assessed using the hazard ratio (HR) and 95% confidence interval (95% CI). Statistical significance was set at *P* < 0.05. Statistical analyses were performed using SPSS for Windows (version 20.0; SPSS, Chicago, IL, USA).

## Results

### Details of patients

**Figure 1:**
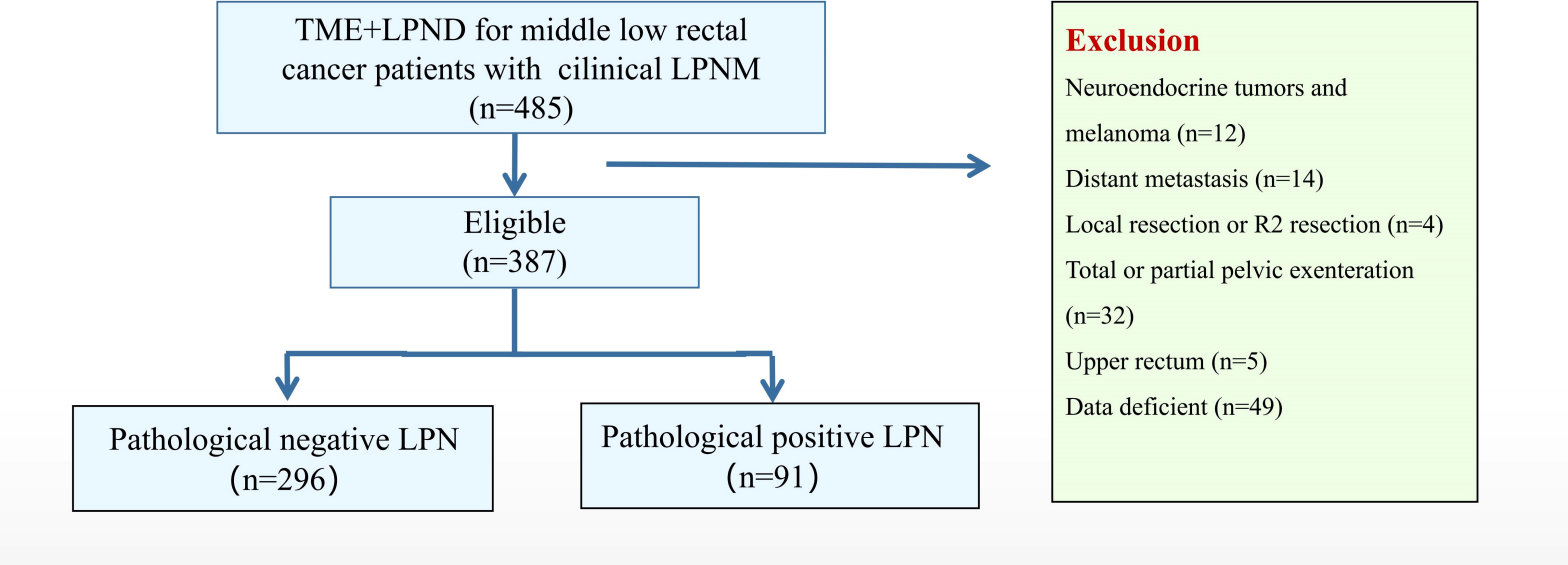
Research flowchart.

In total, 485 patients were identified, of whom 98 were excluded (12 patients with a neuroendocrine tumor or melanoma, 14 with distant metastases, 4 who underwent local resection, 32 who underwent total pelvic exenteration, 5 with upper rectal tumor, and 49 with insufficient data). Finally, 387 patients were included and classified into the positive (n = 91) and negative (n = 296) LPN groups based on the pathological results (**Figure 1**). The enrolled patients were mainly male (61.0%), with an average age of 57.2 years. The CEA level (42.9% vs. 28.0%, *P* = 0.008) in the positive LPN group was significantly higher than that in the negative LPN group. Mucinous and signet adenocarcinoma (27.5% vs. 8.8%, *P* < 0.001) and poor differentiation/non-differentiation (44.0% vs. 26.0%, *P* = 0.002) were more common in the positive LPN group. The proportion of T3–T4 (87.9% vs. 73.0%, *P* = 0.003) and N1–N2 (74.8% vs. 34.5%, *P* < 0.001) in the positive LPN group was significantly higher than that in the negative LPN group. Additionally, patients in the positive LPN group were more likely to develop perineural (51.1% vs. 35.5%, *P* = 0.003) and lymphatic invasion (51.6% vs. 25.0%, *P* < 0.001); moreover, a higher proportion of patients in the positive LPN group received adjuvant chemotherapy (86.8% vs. 58.8, *P* < 0.001). There were no statistically significant differences between the two groups regarding sex, age, body mass index (BMI), distance from the anal verge, preoperative treatment, surgical approach, type of operation, duration of surgery, estimated blood loss, grade 1-2 postoperative complications, and grade 3-5 postoperative complications (*P* > 0.05) (**Table 1**).

**Table 1:** The baseline data and clinicopathological characteristics.

Variables	Positive LPN (n = 91)	Negative LPN (n = 296)	*P*
Age (years, mean ± SD)	57.4 ± 12.2	57.2 ± 10.9	0.900
Sex			0.714
Male	54 (59.3)	182 (61.5)	
Female	37 (40.7)	114 (38.5)	
BMI (kg/m^2^, mean ± SD)	23.9 ± 3.1	24.2 ± 3.4	0.453
Distance from anal verge (cm, mean ± SD)	4.5 ± 2.5	4.8 ± 2.3	0.348
CEA level(ng/ml)			0.008
≥5	39 (42.9)	83 (28.0)	
<5	52 (57.1)	213 (72.0)	
Surgical approach			0.240
Open	21 (23.1)	87 (29.4)	
Laparoscopic	70 (76.9)	209 (80.6)	
Histology			<0.001
Adenocarcinoma	66 (72.5)	270 (91.2)	
Mucinous/signet	25 (27.5)	26 (8.8)	
Differentiated degree			0.002
Well	0 (0)	8 (2.7)	
Moderate	51 (56.0)	211 (71.3)	
Poor/non-differentiated	40 (44.0)	77 (26.0)	
pT stage			0.003
T_0_ -T_2_	11 (12.1)	80 (27.0)	
T_3_-T_4_	80 (87.9)	216 (73.0)	
pN stage (mesorectal LN)			<0.001
N_0_	23 (25.3)	194 (65.5)	
N_1_	45 (49.5)	63 (21.3)	
N_2_	23 (25.3)	39 (13.2)	
Perineural invasion	48 (51.1)	105 (35.5)	0.003
Lymphatic invasion	47 (51.6)	74 (25.0)	<0.001
Type of operation			0.073
Low anterior resection	44 (48.4)	158 (53.4)	
Abdominoperineal resection	39 (42.9)	133 (44.9)	
Hartmann procedure	8 (8.7)	5 (1.7)	
Operative time, median (range) min	280 (140-742)	258 (135-600)	0.409
Estimated blood loss, median (range) mL	100 (10-200)	100 (10-700)	0.566
Postoperative complications (Grade 1-2)	10 (11.0)	33 (11.1)	0.966
Postoperative complications (Grade 3-5)	8 (8.8)	23 (7.8)	0.754
Mortality	1 (1.1)	1(0.3)	0.415
Postoperative hospital stay, median (range) days	12 (5-52)	10 (4-64)	0.367
Adjuvant therapy	79 (86.8)	174 (58.8)	<0.001

LPN, lateral pelvic lymph node; BMI, body mass index; ASA, American Society of Anesthesiologists; CEA, carcinoembryonic antigen.

### Prognostic factors of LPND

**Figure 2:**
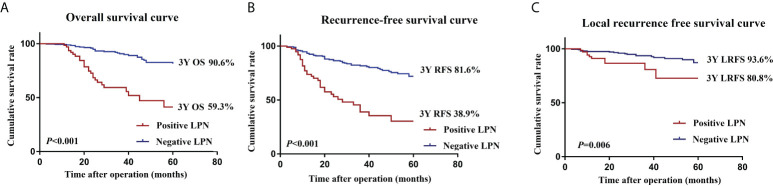
OS **(A)**, RFS **(B)**, and LRFS **(C)** curves of patients in the positive LPN and negative LPN groups. *OS*, overall survival; *RFS*, recurrence-free survival; *LRFS*, local recurrence-free survival; *LPN*, lateral pelvic lymph node.

The mean follow-up period was 41.2 months; 71 of 387 patients died, and 96 had LR or distant metastases. The 3-year OS (90.6 vs. 59.3%, *P <*0.001), 3-year RFS (81.6 vs. 38.9%, *P <*0.001), and 3-year LRFS (93.6 vs. 80.8%, *P* = 0.006) were significantly worse in the positive LPN group than in the negative LPN group (**Figures 2A–C**).

The predictors of the OS were CEA levels, histology, differentiation degree, lymphatic invasion, pN stage, and pathological LPN metastasis (*P* < 0.05). The RFS was significantly affected by preoperative CEA levels, histology, differentiation degree, pT stage, pN stage, pathological LPN metastasis, and adjuvant therapy (*P* < 0.05). The LRFS was significantly affected by lymphatic invasion, preoperative pN stage, and pathological LPN metastasis (*P* < 0.05). Multivariate analysis revealed that pN stage and pathological LPN metastasis were independent prognostic factors for both the OS and RFS (**Tables 2**, **3**), while perineural invasion was an independent prognostic factor for LRFS (**Table 4**).

**Table 2:** Univariate and multivariate regression analyses of overall survival in 387 patients with clinical LPN metastasis who underwent TME+LPND.

Variables	Overall survival			
	Univariate analysis		Multivariate analysis	
	HR (95%CI)	*P*	HR (95%CI)	*P*
Sex: male/female	0.59 (0.35–1.02)	0.058		
Age at operation (≥65/<65 years)	0.79 (0.39–1.56)	0.491		
CEA level (>5/≤5 ng/L)	1.79 (1.03–3.12)	0.038	1.32 (0.75-2.33)	0.338
Distance from anal verge (>5/≤5 cm)	0.55 (0.29–1.05)	0.069		
Operative type: laparoscopic/open	0.93 (0.53–1.63)	0.800		
LPND (Bilateral/Unilateral)	1.36 (0.79–2.35)	0.267		
Histology (Mucinous or signet/adenocarcinoma)	2.95 (1.33-8.27)	0.028	1.42 (0.50-5.89)	0.430
Differentiated degree (Poor or non-differentiated/moderate or well)	3.22 (1.29–7.11)	0.011	1.54 (0.54-4.38)	0.363
Lymphatic invasion (yes/no)	3.63 (1.44–9.14)	0.006	2.13 (0.74-6.14)	0.163
Perineural invasion (yes/no)	2.34 (0.96–5.72)	0.063		
Circumferential Resection Margin (yes/no)	1.45 (0.90-6.21)	0.256		
pT stage (T3–T4/T1-T2)	1.68 (0.86–3.27)	0.126		
pN stage (mesorectal LN)				
N0	–	–	–	–
N1	3.65 (1.65–8.07)	0.001	2.53 (1.12-5.75)	0.026
N2	7.19 (3.35–15.41)	<0.001	3.92 (1.73-8.86)	0.001
Pathological LPN metastasis (yes/no)	4.97 (2.87–8.58)	<0.001	3.27 (1.80-5.93)	<0.001
Grade 3-5 postoperative complication (yes/no)	2.20 (0.88–3.61)	0.114		
Adjuvant therapy (yes/no)	3.32 (0.95-4.92)	0.097		

LPN, lateral pelvic lymph node; TME, total mesorectal excision; CEA, carcinoembryonic antigen; LPND, LPN dissection.

**Table 3:** Univariate and multivariate regression analyses of recurrence-free survival in 387 patients with clinical LPN metastasis who underwent TME+LPND.

Variables	Recurrence-free survival			
	Univariate analysis		Multivariate analysis	
	HR (95%CI)	*P*	HR (95%CI)	*P*
Sex: male/female	0.79 (0.51–1.23)	0.297		
Age at operation (≥65/<65 years)	0.78 (0.46–1.33)	0.366		
CEA level (>5/≤5 ng/L)	1.57 (1.01–2.45)	0.049	1.19 (0.76–1.89)	0.441
Distance from anal verge (>5/≤5 cm)	0.73 (0.45–1.19)	0.203		
Operative type: laparoscopic/open	1.13 (0.72–1.79)	0.593		
LPND (Bilateral/Unilateral)	0.90 (0.57–1.42)	0.641		
Histology (Mucinous or signet/adenocarcinoma)	2.04 (1.43-5.28)	0.037	1.14 (0.74-2.76)	0.483
Differentiated degree (Poor or non-differentiated/moderate or well)	2.35 (1.21–4.33)	0.018	1.82 (0.78–4.25)	0.224
Lymphatic invasion (yes/no)	1.62 (0.80–3.28)	0.180		
Perineural invasion (yes/no)	1.84 (0.94–3.58)	0.075		
Circumferential Resection Margin (yes/no)	1.52 (0.92-4.76)	0.182		
pT stage (T3–T4/T1-T2)	1.73 (1.05–2.99)	0.048	0.86 (0.46–1.60)	0.630
pN stage (mesorectal LN)				
N0	–	–	–	–
N1	3.16 (1.76-5.68)	<0.001	2.73 (1.45–5.16)	0.002
N2	5.44 (3.08-9.63)	<0.001	3.91 (2.00–7.64)	<0.001
Pathological LPN metastasis (yes/no)	3.99 (2.56-6.22)	<0.001	2.84 (1.75–4.63)	<0.001
Grade 3-5 postoperative complication (yes/no)	1.37 (0.81-2.93)	0.352		
Adjuvant therapy (yes/no)	2.45 (1.22-7.32)	0.042	2.87 (0.65-9.32)	0.752

LPN, lateral pelvic lymph node; TME, total mesorectal excision; CEA, carcinoembryonic antigen; LPND, LPN dissection.

**Table 4:** Univariate and multivariate regression analyses of local recurrence-free survival in 387 patients with clinical LPN metastasis who underwent TME+LPND.

Variables	Local recurrence-free survival			
	Univariate analysis		Multivariate analysis	
	HR (95%CI)	*P*	HR (95%CI)	*P*
Sex: male/female	0.97 (0.46-2.05)	0.927		
Age at operation (≥65/<65 years)	0.84 (0.34-2.05)	0.695		
CEA level (>5/≤5 ng/L)	1.15 (0.54-2.49)	0.714		
Distance from anal verge (>5/≤5 cm)	0.40 (0.15-1.04)	0.060		
Operative type: laparoscopic/open	2.29 (0.95-5.52)	0.067		
LPND (Bilateral/Unilateral)	1.32 (0.63-2.77)	0.460		
Histology (Mucinous or signet/adenocarcinoma)	2.04 (0.85-9.42)	0.134		
Differentiated degree (Poor or non-differentiated/moderate or well)	2.41 (0.90-6.42)	0.079		
Lymphatic invasion (yes/no)	2.16 (0.76-6.20)	0.151		
Perineural invasion (yes/no)	3.11 (1.13-8.62)	0.029	3.75 (1.28-10.96)	0.016
Circumferential Resection Margin (yes/no)	3.87 (0.97-8.15)	0.094		
pT stage (T3–T4/T1-T2)	1.61 (0.65-3.96)	0.300		
pN stage (mesorectal LN)				
N0	–	–	–	–
N1	3.16 (1.76-5.68)	0.138	1.18 (0.38-3.70)	0.778
N2	1.98 (0.80-4.88)	0.027	0.57 (0.13-2.52)	0.460
Pathological LPN metastasis (yes/no)	2.85 (1.30-6.24)	0.009	2.42 (0.77-7.64)	0.132
Grade 3-5 postoperative complication (yes/no)	1.25 (0.78-2.63)	0.452		
Adjuvant therapy (yes/no)	2.13 (0.82-5.96)	0.189		

LPN, lateral pelvic lymph node; TME, total mesorectal excision; CEA, carcinoembryonic antigen; LPND, LPN dissection.

### Postoperative recurrence pattern

**Table 5** shows a flowchart with details of up to 3 years of recurrence after the surgery in both groups. In total, 42 patients (42/91, 46.2%) with positive LPN relapsed, 12 (12/91, 13.2%) experienced LR, and 35 (35/91, 38.5%) had distant recurrence. Further, 54 (54/296, 18.2%) patients with negative LPN showed relapse: 18 (18/296, 6.1%) experienced LR and 46 (46/296, 15.5%) had distant recurrences. The proportion of local recurrence in overall recurrence was similar in positive LPN and negative LPN groups (12/42, 28.6% vs. 18/54, 33.3%).

**Table 5:** Recurrence rate of local and distant metastasis.

Recurrence pattern	Positive LPN (n = 91)	Negative LPN (n = 296)
Overall recurrence	42 (46.2)	54 (18.2)
Local recurrence	12 (13.2)	18 (6.1)
Central pelvic	8 (8.8)	10 (3.4)
Lateral pelvic	3 (3.3)	6 (2.0)
Central pelvic+Lateral pelvic	1 (1.1)	2 (0.7)
Distant recurrence	35 (38.5)	46 (15.5)
Lung	10 (11.0)	17 (5.7)
Liver	6 (6.6)	8 (2.7)
Bone	3 (3.3)	5 (1.7)
Peritoneum	2 (2.2)	2 (0.7)
Lymph nodes	3 (3.3)	2 (0.7)
Lung+liver	5 (5.5)	8 (2.7)
Liver+Bone	1 (1.1)	1 (0.3)
Liver+Peritoneum	2 (2.2)	0 (0)
Liver+Bone+Lung	3 (3.3)	0 (0)

LPN, lateral pelvic lymph node.

### Prognostic factors of LPN metastasis

**Table 6** shows the distribution of the number of LPNs in patients with LPN metastases. Considering LPNs ≥ 3 as the cut-off value, the AUC for prognosis in patients with LPN metastasis was 0.634, showing good concordance. Therefore, we considered LPNs ≥ 3 as the cut-off value for predicting prognosis in patients with LPN metastasis in the present study.

**Table 6:** The distribution of the number of LPNs in 91 patients with LPN metastases.

Number of positive LPN	Number of patients	HR	95%CI	*P*	AUC
LPNs ≥2	37/54	1.46	0.75-2.86	0.265	0.569
LPNs ≥3	16/75	2.09	1.02-4.30	0.044	0.634
LPNs ≥4	15/76	1.94	0.92-4.07	0.080	0.620
LPNs ≥5	9/82	2.77	1.21-6.36	0.016	0.600
LPNs ≥6	5/86	1.43	0.43-4.68	0.559	0.543
LPNs ≥7	3/88	2.04	0.48-8.56	0.332	0.529

LPN, lateral pelvic lymph node; HR, hazard ratio; AUC, area under the curve

**Table 7** shows the univariate and multivariate regression analyses results of the 91 patients with pathological LPN metastasis. Univariate analysis identified LPN metastasis to the common iliac and external iliac arteries, LPNs ≥ 3, and bilateral LPN metastasis as predictors of the OS; the predictors of RFS were open LPND, N2 stage, LPN metastasis to the common iliac and external iliac arteries, LPNs ≥3, and bilateral LPN metastasis (*P* < 0.05). Multivariate analysis revealed that the LPN metastasis to the common iliac and external iliac arteries was an independent prognostic factor for both the OS (HR, 4.74; 95% CI, 1.74−12.90; *P* = 0.002) and RFS (HR: 2.70; 95% CI, 1.16−6.29; *P* = 0.021).

**Table 7:** Univariate and multivariate regression analyses of 91 patients with pathological LPN metastasis.

Variables	Overall survival				Recurrence-free survival			
	Univariate analysis			Multivariate analysis		Univariate analysis		Multivariate analysis	
	HR (95%CI)	*P*	HR (95%CI)	*P*	HR (95%CI)	*P*	HR (95%CI)	*P*
Sex: male/female	0.72 (0.32-1.61)	0.428			0.90 (0.46-1.77)	0.760		
Age at operation (≥65/<65years)	0.48 (0.16-1.39)	0.175			0.70 (0.33-1.50)	0.362		
CEA level(>5/≤5 ng/L)	1.66 (0.70-3.94)	0.247			1.99 (0.99-4.01)	0.053		
Histology (Mucinous or signet/adenocarcinoma)	1.89 (0.49-8.84)	0.652			1.39 (0.42-9.68)	0.542		
Differentiated degree (Poor or non-differentiated/moderate or well)	1.73 (0.45-11.51)	0.579			1.47 (0.62-10.68)	0.433		
Operative type: laparoscopic/open	0.47 (0.21-1.04)	0.063			0.44 (0.23-0.87)	0.017	0.57 (0.27-1.21)	0.145
Lymphatic invasion (yes/no)	4.36 (0.72-35.89)	0.164			1.76 (0.88-5.02)	0.131		
Perineural invasion (yes/no)	2.74 (0.45-16.67)	0.275			2.26 (0.56-9.14)	0.254		
Circumferential Resection Margin (yes/no)	1.44 (0.91-5.82)	0.134			1.70 (0.85-7.35)	0.381		
pT stage (T3-T4/T0-T2)	0.83 (0.28-2.42)	0.726			0.49 (0.20-1.19)	0.115		
pN stage (mesorectal LN)								
N0	–	–			–	–		
N1	2.87 (0.36-22.99)	0.321			2.52 (0.56-11.30)	0.229	2.17 (0.48-9.95)	0.318
N2	5.92 (0.78-44.93)	0.085			4.62 (1.08-19.77)	0.039	3.28 (0.72-14.96)	0.125
LPN metastasis (obturator or internal iliac artery/other)	6.10 (2.54-14.68)	<0.001	4.74 (1.74-12.90)	0.002	2.74 (1.25-6.02)	0.012	2.70 (1.16-6.29)	0.021
Number of LPN metastasis (≥3/< 3)	2.69 (1.18-6.18)	0.019	1.51 (0.59-3.82)	0.391	2.09 (1.02-4.30)	0.044	1.10 (0.48-2.52)	0.828
LPN metastasis (Bilateral/Unilateral)	3.16 (1.16-8.60)	0.024	1.33 (0.44-4.07)	0.617	2.17 (0.90-5.27)	0.086		
Adjuvant therapy (yes/no)	0.87 (0.36-2.10)	0.753						

LPN, lateral pelvic lymph node; CEA, carcinoembryonic antigen; HR, hazard ratio

**Figure 3:**
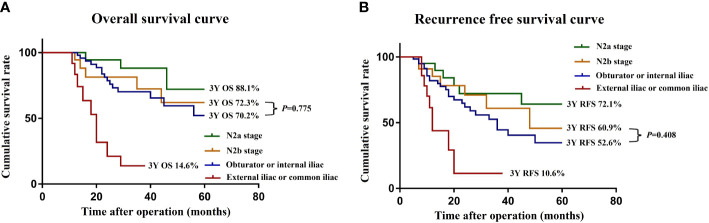
OS **(A)** and RFS **(B)** curves of patients with LPN metastasis and N2 stage after subgroup analysis. *OS*, overall survival; *RFS*, recurrence-free survival.

To further clarify the prognostic significance of the location of the LPN metastasis, we screened 89 patients at stage N2 from the negative LPN group and subdivided them into N2a stage (4−6 regional lymph node metastases) and N2b stage (≥7 regional lymph node metastases) according to the AJCC tumor staging system. The 3-year OS (and the 3-year RFS) rates of the N2a stage, N2b stage, LPN metastasis to the obturator or internal iliac, and LPN metastasis to the common iliac and external iliac arteries were 88.1% (72.1%), 72.3% (60.9%), 70.2% (52.6%), and 14.6% (10.6%), respectively (**Figures 3A, B**). The OS (*P* = 0.775) and RFS (*P* = 0.408) were not significantly different between patients with the LPN metastasis to the obturator or internal iliac artery and at the N2b stage.

## Discussion

Due to the complexity of the anatomical location of the pelvic lateral wall and the difficulty of preserving the pelvic nerve plexus, the execution of LPND has high technical requirements for surgeons. Our study showed that the median operative time was 265 min, the median estimated blood loss was 100 mL, and the rate of grade 3-5 postoperative complication rate was 8.0% (31/387). Ogura et al. reported on 107 patients who underwent laparoscopic LPND + TME and showed that the mean operation duration and blood loss were 461 min and 115 mL, respectively. Furthermore, no significant difference was found in postoperative complications, consistent with our results (18). Therefore, we suggest that LPND is safe and feasible in institutions with experienced surgeons.

The lateral pelvis is a common site of postoperative recurrence after rectal cancer surgery (19), and procedures to suppress the LR are optimized separately in various countries. The standard treatment in Western countries is nCRT, followed by TME; however, recent data suggest that nCRT cannot completely eradicate LPN metastasis and has a high risk of lateral compartment recurrence (18, 20). Therefore, LPND is a crucial and necessary approach to reducing the LR in patients with LPN metastasis. In the present study, although the OS and RFS of patients with LPN metastasis were still poor after LPND, LPN metastasis was not an independent adverse prognostic factor for LRFS after the elimination of confounding factors through multivariate Cox regression analysis. Moreover, by analyzing the recurrence patterns, we found that the same proportion of local recurrence accounted for overall recurrence in both groups. Therefore, we believe that the local control effect of LPND was satisfactory. However, some patients with LPN metastasis showed potential micrometastasis at diagnosis, which is an important cause of frequent distant metastasis after the surgery. Strengthening systemic adjuvant chemotherapy is needed to improve patient survival.

The survival benefits of LPND for patients with LPN metastasis depend on whether the local metastases can be cured by resection or if there is distant metastases that cannot be controlled locally. Previous studies confirmed that the location (5, 13, 21) and the number of LPN metastases (19, 22–24) are factors that affect the efficacy of LPND in patients with LPN metastases. In the present study, multivariate analysis demonstrated that lymph node metastasis to the common iliac or external iliac artery was an independent adverse prognostic factor for patients with LPN metastasis, with low 3-year OS and RFS rates. Moreover, we further evaluated the prognostic significance of LPND in patients with LPN metastasis, and the results showed that the 3-year OS and 3-year RFS of patients with internal iliac or obturator lymph node metastases were comparable with those at the N2b stage. Kanemitsu et al. conducted a retrospective study involving 1,191 patients from two high-volume centers in Japan, and the results showed that the survival benefits from LPND of the internal iliac and obturator areas were comparable to those obtained through dissection of the superior rectal artery area (13). Similarly, Morohashi et al. reported that lymph node metastasis outside the internal iliac and obturator lymph node area was an independent prognostic risk factor for patients with LPN metastasis (HR: 2.70; 95% CI, 1.20–6.08; *P* = 0.016) (21). The above literature is consistent with our study results, suggesting that external iliac or common iliac lymph node metastasis could be regarded as a systemic disease and that prognosis cannot improve even with dissection But that leads to increased urinary and sexual dysfunction; thus, the systemic comprehensive therapy should be strengthened to replace LPND. Additionally, patients with internal iliac or obturator lymph node metastases could obtain survival benefits after LPND, and we considered that they had regional lymph node metastases (N2b stage) that can be cured by LPND.

The results of this study suggest that LPND is invalid and undesirable because only 23.5% (91/387) of patients have pathology confirming LPN metastasis, which leads to excessive unnecessary LPND. The treatment for LPN metastasis is essentially multidisciplinary and comprehensive. In recent years, nCRT and LPND have been integrated and become complementary; they form a relatively scientific treatment system. Currently, in China, patients with clinical evidence of LPN metastasis are recommended to undergo nCRT first, followed by selective LPND. We have gradually established standardized and practical indications for selective LPND after nCRT, and we suggest that patients with LPN diameter ≥ 7 mm after nCRT or poor differentiation should be offered LPND after nCRT; moreover, patients without risk factors can adopt a watch-and-wait treatment strategy after nCRT to avoid over-treatment (17). Additionally, the presence of distant metastases showed a dominant recurrence pattern in this study. We hypothesized that nCRT incompletely eliminated micrometastasis, highlighting the importance of strengthening adjuvant chemotherapy. However, poor compliance and a high risk of complications hinder the progression of postoperative adjuvant chemotherapy. Therefore, postoperative adjuvant chemotherapy may be considered for controlling toxicity and chemotherapy before, during, and after radiotherapy (totally neoadjuvant therapy), or to replace radiotherapy with intensive chemotherapy (25, 26). These findings still need to be confirmed by prospective randomized controlled studies in the future.

This study had several limitations. First, besides the limited number of participants, the retrospective multicenter nature of this study has inherent selection bias and heterogeneity of patients and treatments. Second, the decision to implement LPND is based on preoperative evaluation, but the prognostic factor obtained in this study was the location of LPN based on pathological assessment, and this information is unavailable to the surgeon before surgery. However, with continuous development and improvement in radiology, preoperative MRI can accurately assess the short diameter, location, quantity, heterogeneity, and other imaging characteristics of the metastatic LPN. Therefore, we believe that the findings of this study can still provide some guidance for clinical practice. Third, the average BMI of all patients in this study was 24 kg/m^2^, which was significantly lower than that of patients in Western countries. Rectal dissection and LPND may be more technically difficult in obese patients; thus, a large difference in BMI would undermine the generalizability of this study. Finally, the mean follow-up time of the whole study was only 41.2 months, which is insufficient for adequately assessing the 5-year survival outcomes.

## Conclusion

The results of this study suggest that although LPND may be effective in controlling the LR in patients with LPN metastasis, systemic spread and metastases frequently develop postoperatively. Patients with LPN metastases limited to the internal iliac and obturator regions appear to achieve a long-term survival benefit from LPND, and their prognoses may be comparable to those of patients at the N2b stage. Further metastasis to the external iliac or common iliac region should be considered a systemic disease, and LPND should be avoided.

## Data availability statement

The raw data supporting the conclusions of this article will be made available by the authors, without undue reservation.

## Ethics statement

The studies involving human participants were reviewed and approved by Each of the seven participating institutions received local ethical approval for the study and the protocol was registered (NCT04850027) at ClinicalTrials.gov. Informed consent was obtained from all the patient prior to enrollment, while all the procedures of the study were in accordance with the tenets of the Declaration of Helsinki. The patients/participants provided their written informed consent to participate in this study.

## Author contributions

(I) conception and design: QL,YX, and SCZ; (II) administrative support: QL; (III) provision of study materials or patients: JWL, ZL, WF, BF and YCY; (IV) collection and assembly of data: SCZ and JQT; (V) data analysis and interpretation: QL, JQT, and SCZ. All authors read and approved the final.

## Funding

This study received the funding from the National Key Research and Development Program/Prevent and Control Research for Important Non-Communicable Diseases (No. 2019YFC1315705) and the Medicine and Health Technology Innovation Project of the Chinese Academy of Medical Sciences (No. 2017-12 M-1e006).

## Acknowledgments

The study protocol was registered (NCT04850027) at ClinicalTrials.gov. Thanks are due to all members of the Chinese Lateral Node Collaborative Group for their support of this study data and review of this article.

## Conflict of interest

The authors declare that the research was conducted in the absence of any commercial or financial relationships that could be construed as a potential conflict of interest.

## Publisher’s note

All claims expressed in this article are solely those of the authors and do not necessarily represent those of their affiliated organizations, or those of the publisher, the editors and the reviewers. Any product that may be evaluated in this article, or claim that may be made by its manufacturer, is not guaranteed or endorsed by the publisher.

## References

[1] Heald RJ Ryall RDH. Recurrence and survival after total mesorectal excision for rectal cancer. Lancet (1986) 327:1479–82. doi: 10.1016/s0140-6736(86)91510-2 2425199

[2] Taylor FGM Quirke P Heald RJ Moran BJ Blomqvist L Swift IR. Magnetic resonance imaging in rectal cancer European equivalence study group. preoperative magnetic resonance imaging assessment of circumferential resection margin predicts disease-free survival and local recurrence: 5-year follow-up results of the MERCURY study. J Clin Oncol (2014) 32:34–43. doi: 10.1200/JCO.2012.45.3258 24276776

[3] Beets-Tan RGH Lambregts DMJ Maas M Bipat S Barbaro B Curvo-Semedo L. Magnetic resonance imaging for clinical management of rectal cancer: Updated recommendations from the 2016 European society of gastrointestinal and abdominal radiology (ESGAR) consensus meeting. Eur Radiol (2018) 28:1465–75. doi: 10.1007/s00330-017-5026-2 PMC5834554 29043428

[4] Kapiteijn E Marijnen CA Nagtegaal ID Putter H Steup WH Wiggers T. Preoperative radiotherapy combined with total mesorectal excision for resectable rectal cancer. N Engl J Med (2001) 345:638–46. doi: 10.1056/NEJMoa010580 11547717

[5] Akiyoshi T Watanabe T Miyata S Kotake K Muto T Sugihara K. Japanese Society for cancer of the colon and rectum. results of a Japanese nationwide multi-institutional study on lateral pelvic lymph node metastasis in low rectal cancer: is it regional or distant disease?: is it regional or distant disease? Ann Surg (2012) 255:1129–34. doi: 10.1097/SLA.0b013e3182565d9d 22549752

[6] Sugihara K Kobayashi H Kato T Mori T Mochizuki H Kameoka S. Indication and benefit of pelvic sidewall dissection for rectal cancer. Dis Colon Rectum (2006) 49:1663–72. doi: 10.1007/s10350-006-0714-z 17041749

[7] Yano H Moran BJ. The incidence of lateral pelvic side-wall nodal involvement in low rectal cancer may be similar in Japan and the West. Br J Surg (2008) 95:33–49. doi: 10.1002/bjs.6061 18165939

[8] Williamson JS Quyn AJ Sagar PM. Rectal cancer lateral pelvic sidewall lymph nodes: a review of controversies and management: Lateral pelvic sidewall nodes in rectal cancer. Br J Surg (2020) 107:1562–9. doi: 10.1002/bjs.11925 32770742

[9] Emile SH Elfeki H Shalaby M Sakr A Kim NK. Outcome of lateral pelvic lymph node dissection with total mesorectal excision in treatment of rectal cancer: A systematic review and meta-analysis. Surgery (2021) 169:1005–15. doi: 10.1016/j.surg.2020.11.010 33317903

[10] Bosset J-F Calais G Mineur L Maingon P Stojanovic-Rundic S Bensadoun R-J. Fluorouracil-based adjuvant chemotherapy after preoperative chemoradiotherapy in rectal cancer: long-term results of the EORTC 22921 randomised study. Lancet Oncol (2014) 15:184–90. doi: 10.1016/S1470-2045(13)70599-0 24440473

[11] Fujita S Mizusawa J Kanemitsu Y Ito M Kinugasa Y Komori K. Mesorectal excision with or without lateral lymph node dissection for clinical stage II/III lower rectal cancer (JCOG0212): A multicenter, randomized controlled, noninferiority trial. Ann Surg (2017) 266:201–7. doi: 10.1097/sla.0000000000002212 28288057

[12] Hashiguchi Y Muro K Saito Y Ito Y Ajioka Y Hamaguchi T. Japanese Society for cancer of the colon and rectum (JSCCR) guidelines 2019 for the treatment of colorectal cancer. Int J Clin Oncol (2020) 25:1–42. doi: 10.1007/s10147-019-01485-z 31203527 PMC6946738

[13] Kanemitsu Y Komori K Shida D Ochiai H Tsukamoto S Kinoshita T. Potential impact of lateral lymph node dissection (LLND) for low rectal cancer on prognoses and local control: A comparison of 2 high-volume centers in Japan that employ different policies concerning LLND. Surgery (2017) 162:303–14. doi: 10.1016/j.surg.2017.02.005 28366499

[14] Nicholls RJ Zinicola R Haboubi N. Extramural spread of rectal cancer and the AJCC cancer staging manual 8th edition, 2017. Ann Oncol (2019) 30:1394–5. doi: 10.1093/annonc/mdz147 31046085

[15] Clavien PA Barkun J de Oliveira ML Vauthey JN Dindo D Schulick RD. The clavien-dindo classification of surgical complications: five-year experience: Five-year experience. Ann Surg (2009) 250:187–96. doi: 10.1097/SLA.0b013e3181b13ca2 19638912

[16] Wang P Zhou S Zhou H Liang J Zhou Z. Evaluating predictive factors for determining the presence of lateral pelvic node metastasis in rectal cancer patients following neoadjuvant chemoradiotherapy. Colorectal Dis (2019) 21:791–6. doi: 10.1111/codi.14595 30801862

[17] Zhou S Jiang Y Pei W Zhou H Liang J Zhou Z. Neoadjuvant chemoradiotherapy followed by lateral pelvic lymph node dissection for rectal cancer patients: A retrospective study of its safety and indications. J Surg Oncol (2021) 124:354–60. doi: 10.1002/jso.26509 33882149

[18] Ogura A Akiyoshi T Nagasaki T Konishi T Fujimoto Y Nagayama S. Feasibility of laparoscopic total mesorectal excision with extended lateral pelvic lymph node dissection for advanced lower rectal cancer after preoperative chemoradiotherapy. World J Surg (2017) 41:868–75. doi: 10.1007/s00268-016-3762-0 27730352

[19] Kinugasa T Akagi Y Shirouzu K. Benefit of lateral lymph node dissection for rectal cancer: long-term analysis of 944 cases undergoing surgery at a single center (1975-2004). Anticancer Res (2014) 34:4633–9. doi: 10.1016/j.urolonc.2014.05.010 25075111

[20] Ogura A Konishi T Cunningham C Garcia-Aguilar J Iversen H Toda S. Neoadjuvant (chemo)radiotherapy with total mesorectal excision only is not sufficient to prevent lateral local recurrence in enlarged nodes: Results of the multicenter lateral node study of patients with low cT3/4 rectal cancer. J Clin Oncol (2019) 37:33–43. doi: 10.1200/JCO.18.00032 30403572 PMC6366816

[21] Morohashi H Sakamoto Y Miura T Ichinohe D Umemura K Akaishi T. Effective dissection for rectal cancer with lateral lymph node metastasis based on prognostic factors and recurrence type. Int J Colorectal Dis (2021) 36:1251–61. doi: 10.1007/s00384-021-03870-5 PMC8119260 33527145

[22] Ozawa H Nakanishi H Sakamoto J Suzuki Y Fujita S. Prognostic impact of the number of lateral pelvic lymph node metastases on rectal cancer. Jpn J Clin Oncol (2020) 50:1254–60. doi: 10.1093/jjco/hyaa122 32687179

[23] Sato H Maeda K Maruta M Masumori K Koide Y. Who can get the beneficial effect from lateral lymph node dissection for dukes c rectal carcinoma below the peritoneal reflection? Dis Colon Rectum (2006) 49:S3–S12. doi: 10.1007/s10350-006-0699-7 17106812

[24] Fujita S. Incidence and prognosis of lower rectal cancer with limited extramesorectal lymph node metastasis. Int J Colorectal Dis (2014) 29:1077–80. doi: 10.1007/s00384-014-1940-9 24972679

[25] Fokas E Allgäuer M Polat B Klautke G Grabenbauer GG Fietkau R. Randomized phase II trial of chemoradiotherapy plus induction or consolidation chemotherapy as total neoadjuvant therapy for locally advanced rectal cancer: CAO/ARO/AIO-12. J Clin Oncol (2019) 37:3212–22. doi: 10.1200/JCO.19.00308 31150315

[26] Fernández-Martos C Pericay C Losa F García-Carbonero R Layos L Rodríguez-Salas N. Effect of aflibercept plus modified FOLFOX6 induction chemotherapy before standard chemoradiotherapy and surgery in patients with high-risk rectal adenocarcinoma: The GEMCAD 1402 randomized clinical trial: The GEMCAD 1402 randomized clinical trial. JAMA Oncol (2019) 5:1566–73. doi: 10.1001/jamaoncol.2019.2294 PMC6865228 31465088

